# A perspective on epigenomic aging processes in the human brain and their plasticity in patients with mental disorders – a systematic review

**DOI:** 10.1007/s10048-024-00771-x

**Published:** 2024-07-05

**Authors:** Jan Postberg, Michèle Tina Schubert, Vincent Nin, Lukas Wagner, Martina Piefke

**Affiliations:** 1 Clinical Molecular Genetics and Epigenetics, Faculty of Health, Witten/Herdecke University, Alfred-Herrhausen-Str. 50, 58448 Witten, Germany; 2 Centre for Biomedical Education & Research (ZBAF), Witten/Herdecke University, Alfred-Herrhausen-Str. 50, 58448 Witten, Germany; 3 Neurobiology and Genetics of Behavior, Department of Psychology and Psychotherapy, Faculty of Health, Witten/Herdecke University, Alfred-Herrhausen-Str. 50, 58448 Witten, Germany

**Keywords:** Epigenetic aging, Mental disorders, DNA methylation, Brain tissue, Frontal cortex, Schizophrenia, Depression, Epigenetic clocks, Nature vs. nurture, Environmental influences

## Abstract

The debate surrounding nature versus nurture remains a central question in neuroscience, psychology, and in psychiatry, holding implications for both aging processes and the etiology of mental illness. Epigenetics can serve as a bridge between genetic predisposition and environmental influences, thus offering a potential avenue for addressing these questions. Epigenetic clocks, in particular, offer a theoretical framework for measuring biological age based on DNA methylation signatures, enabling the identification of disparities between biological and chronological age. This structured review seeks to consolidate current knowledge regarding the relationship between mental disorders and epigenetic age within the brain. Through a comprehensive literature search encompassing databases such as EBSCO, PubMed, and ClinicalTrials.gov, relevant studies were identified and analyzed. Studies that met inclusion criteria were scrutinized, focusing on those with large sample sizes, analyses of both brain tissue and blood samples, investigation of frontal cortex markers, and a specific emphasis on schizophrenia and depressive disorders. Our review revealed a paucity of significant findings, yet notable insights emerged from studies meeting specific criteria. Studies characterized by extensive sample sizes, analysis of brain tissue and blood samples, assessment of frontal cortex markers, and a focus on schizophrenia and depressive disorders yielded particularly noteworthy results. Despite the limited number of significant findings, these studies shed light on the complex interplay between epigenetic aging and mental illness. While the current body of literature on epigenetic aging in mental disorders presents limited significant findings, it underscores the importance of further research in this area. Future studies should prioritize large sample sizes, comprehensive analyses of brain tissue and blood samples, exploration of specific brain regions such as the frontal cortex, and a focus on key mental disorders. Such endeavors will contribute to a deeper understanding of the relationship between epigenetic aging and mental illness, potentially informing novel diagnostic and therapeutic approaches.

**Supplementary Information:**

The online version contains supplementary material available at 10.1007/s10048-024-00771-x.

## Introduction

**Fig. 1:**
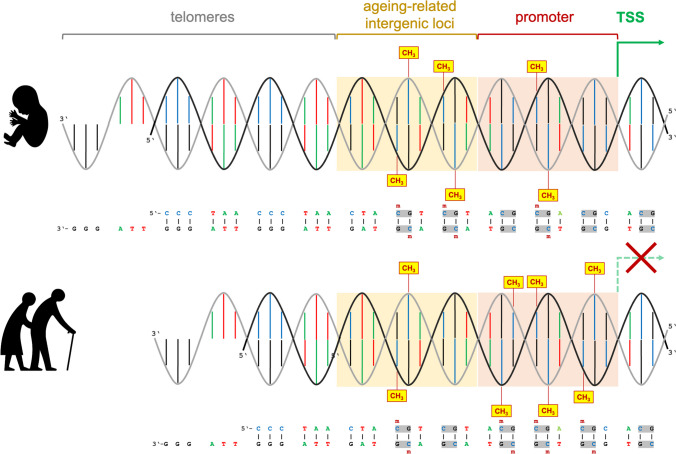
The molecular aging process in cells affects the genome integrity and the DNAm signature. The ends of chromosomes are organized as telomeric complexes. Telomeres comprise of [TTAGGG]_n_ tandem repeats of double-stranded DNA and protruding single-stranded 3’-overhangs. Due to the absence of telomerase activity in healthy somatic cells gradual telomere shortening with each cell cycle is an age-dependent process, which limits the maximum number of cell divisions, thus providing a protective anti-proliferative mechanism. Telomere length is correlated with biological aging. However, their length of origin at the beginning of ontogeny seems to be heterogeneous between sexes and ethnicities [59], which complicates calibration when longitudinal measurements are not available. Whereas genome-wide studies on DNAm signatures in monozygotic twins suggest a high level of persistence of early genome programming [60], subtle changes in numerous genetic loci are observed at the same time, which correlate with biological age. These form the basis for the concept of so-called epigenetic clocks [23–25]. Such age-related DNAm variations can take place in intergenic regions (yellow shaded) as well as in the vicinty of gene promoters (red shaded), where they can take influence on gene regulation. The frequently used ‘Horvath clock’ is, in combination with Illumina’s Infinium HumanMethylome450 BeadChips, calibrated for 353 CpG sites, which can be associated with genes, promoters, long-range regulatory elements or intergenic DNA with no known function [26, 27]. The illustration exemplarily shows an age-related hypermethylated intergenic region as well as a hypomethylated gene promoter during fetal growth that cis-activates mRNA synthesis from adjacent transcription start site (TSS). Below are shown the results of senescence, whereafter the same intergenic region became hypomethlylated and the promoter became hypermethylated. Promoter hypermethylation resulted in the suppression of mRNA synthesis from the adjacent TSS

The nature-nurture debate still holds unanswered questions in the neurosciences and psychology and is relevant for understanding the etiology of mental illness. As a connector between disposition and environment, epigenetics may be capable of answering some of these questions, since the epigenome is the molecular switchboard that mediates between environmental influences and the interpretation of genetic information in the different cell types of the body [1]. However, deep neuroepigenetic insights in humans are complicated by the fact that the plasticity of the epigenome—unlike genetic variation —is variable between the different somatic cell types of an organism and over time. The need for invasive sampling procedures is therefore an almost insurmountable hurdle for human neuroepigenetics unless there is a strong clinical indication. Nevertheless, in order to gain some insights, the field has recently focused on such molecular markers through which inferences can be made about other body cell types from biomaterials obtained in a less invasive manner (e.g. leukocytes, buccal mucosa cells, urine). In particular, changes over time in the epigenome (DNA methylation [DNAm]) or chromosomes (telomere length) are documented in the literature to measure the biological age of a sample (Fig. 1). In particular, epigenetic clocks are theoretically suitable for detecting discrepancies between the measured biological age of a sample and the chronological age of a sample in order to detect indications of an accelerated or decelerated aging process that might be responsive to environmental influences.

Every human being ages. The most obvious manifestations of this process include skin changes, hair greying and/or loss of hair, changes in vision, bone density and dental appearance as well as reductions in physical performance, and cognitive capacities [2, 3]. In healthy somatic cells, the maximum number of cell divisions is limited by an aging process through molecular programming. Accompanying symptoms of cellular aging include genomic instability, telomere shortening, epigenetic changes, mitochondrial dysfunction, stem cell depletion, altered intercellular communication and immune senescence [4]. Premature aging is a condition in which biological hallmarks of the aging process do not match the changes expected with respect to the chronological age. This can be caused by various environmental influences. For example, strong sunlight exposure, especially UV light, can lead to increased DNA damage in skin cells and visible changes in the skin. Other environmental triggers can be smoking, unhealthy diet, alcohol consumption, lack of sleep and negative stress. In rarer cases, there are genetic diseases that can massively accelerate the natural aging process, such as Bloom syndrome, Werner syndrome, dyskeratosis congenita or Hutchison Gilford progeria syndrome[5, 6].

Interestingly, a variety of mental illnesses are symptomatically reminiscent of premature aging. Noticeable are motor, sensory, and cognitive impairments as well as alterations in cardiovascular and metabolic markers, and cellular biomarkers [7, 8]. People with mental illness also have an increased risk of mortality [9]. To study the correlations and cause-and-effect mechanisms between biological aging and mental illness, reliable age markers are required.

Molecular alterations dependent on age impact the epigenome’s plasticity on different molecular levels, such as DNA cytosine methylation, histone post-translational modifications as well as the 3D organization of the DNA in the nucleus and how it changes over time [10–12]. Epigenomes differ between the distinct somatic cell types of an organism, whereas their genomes do not. The epigenome is the sum of chemical compounds that modify or mark the genome in such a way that the information it contains can be interpreted in different cell types and at different time points, adapted to the requirements at hand [1].

The most direct level of epigenome modification is DNA cytosine methylation. This involves the covalent transfer of a methyl group to the cytosine within CpG dinucleotides through DNA methyl transferases (DNMTs) [13]. DNAm is – among other processes – involved in gene regulation, cell differentiation and development, X chromosome inactivation, and genetic imprinting [14–18] as well as in cognitive processes such as learning and memory formation [19]. Interestingly, environmental influences can affect DNAm signatures relevant for human behavior [20]. DNAm changes across the lifespan and forms age-typical patterns at several genomic loci [21, 22]. In the last decade, methods have been developed that exploit DNAm patterns to measure the epigenetic age of a specimen. To date, several models for ‘epigenetic clocks’ exist [23–25], differing in for example, the type of tissue/cells being used and the number of CpG sites examined. Frequently used is Horvarth’s epigenetic clock, which uses 353 CpG positions to determine an epigenetic age which correlates to 0.96 with chronological age. This model is reported to fit for 51 tissue types [26, 27]. Other commonly used clocks focus on peripheral tissue such as blood or saliva [28, 29]. Epigenetic clocks are outstandingly interesting since, in contrast to non-repairable DNA damage that impairs genome integrity and can induce mutations, changes in the epigenome are fundamentally reversible. Because they are at the same time apparently responsive to some environmental influences, it may be hypothesized that the aging-dependent DNAm signature could be influenced bidirectionally and thus be a biomarker for accelerated but also for decelerated aging processes.

In the field of neuroscience, there is evidence that stress and nutrition may slow down or accelerate epigenetic aging. Mental illnesses also appear to influence epigenetic age. However, so far these studies have been performed primarily on peripheral tissue and different studies come to inconsistent conclusions [25]. Although peripheral tissues, such as blood, prove to be convenient due to their easy accessibility, cortical mental illnesses and brain tissue aging samples are the most practical for studying DNAm in mental disorders [30]. A limiting factor for research is that they are usually not available for most conditions and healthy control groups in vivo. Given these limitations, we must carefully weigh up whether peripheral tissue can be used as a proxy for brain tissue. There are indications that epigenetic aging markers of peripheral blood and brain may exhibit rather small, and sometimes even no correlations, at least in older individuals [31]. Notably, when considering either physiological or psychological correlates of accelerated epigenetic aging in blood cells, results for physiological outcomes are consistent (e.g. lung function), but results for psychological outcomes are very inconsistent (e.g. mental illness) [25]. In sum, the reliability of epigenetic clock data obtained from peripheral tissues with respect to their applicability in psychology seems questionable. Therefore, in this review, we interrogate current state of knowledge about the relationship between mental disorders and epigenetic age in the brain.

## Methods

The review is based on the PRISMA guidelines.

### Inclusion criteria

#### PICO criteria

The PICO (population, intervention/exposure, comparison, outcome) criteria were used to specify the research question.

##### Population

No specific inclusion criteria were defined for demographic or other variables.

##### Intervention/exposure

individuals with mental illnesses.

##### Comparison

The control group included people without mental illness.

##### Outcome

The epigenetic age in the brain, and/or a difference between the epigenetic age in the brain between people with and without mental illnesses.

#### Types of studies

The studies had to be published in German or English language in international peer-reviewed scientific journals. There were no restrictions regarding the study design. Due to the young age of the research field, no restrictions were made for publication years.

#### Epigenetic clock

Following Oblak et al.’s definition of epigenetic clock [25], studies were included that determined epigenetic age based on methylation patterns at CpG dinucleotides.

### Search strategy

**Fig. 2:**
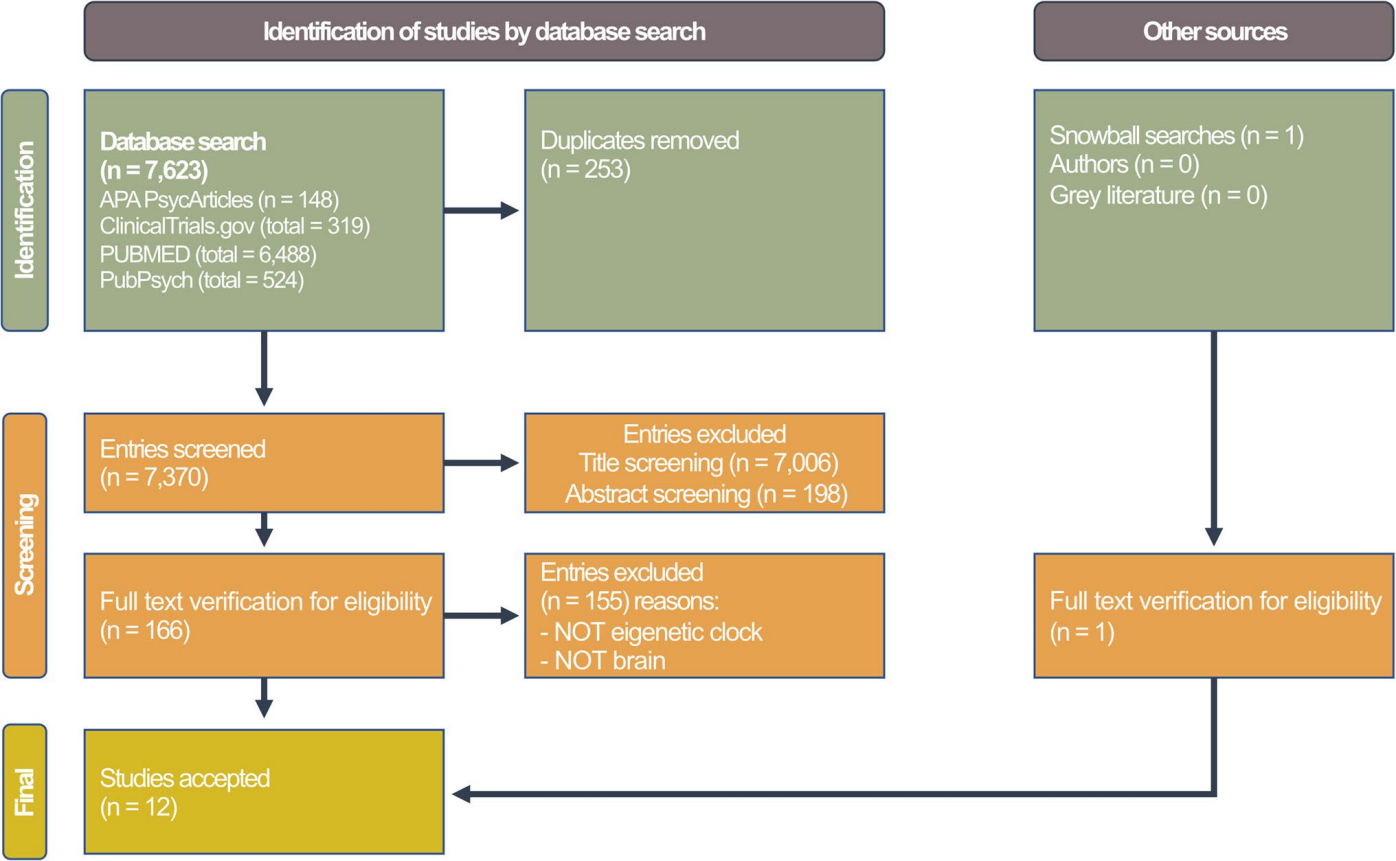
Selection process of relevant studies. A comprehensive literature search across multiple databases and gray literature sources yielded 7,623 titles. After meticulous screening and application of inclusion criteria, 12 studies meeting the criteria were identified for analysis

Using a literature search of the EBSCO (Psychology and Behavioral Sciences Collection), Pubmed, APA PsycArticles, PubPsych (Psyndex, NARCIS, PASCAL, ERIC), and ClinicalTrials.gov databases, relevant studies were selected that had been published by February 21, 2022. A search using the keywords “epigenetic clock” OR “epigenetic age” OR “epigenetic aging” OR “age acceleration” OR “age deceleration” OR “DNAm age” OR “dna methylation” AND “brain” yielded 7,623 titles. Studies that did not involve epigenetic clocks, mental disorders, or did not directly target the human brain were excluded. Gray literature was searched, and relevant authors were contacted. Using the snowball method, references of the found original articles and relevant reviews were checked for any further studies. A total of 12 studies were identified that matched the criteria. Figure 2 illustrates the selection process using a flowchart.

### Data synthesis

Information was extracted on study designs, subjects, mental disorders, methods of data collection, sampled tissues, DNA extraction, genetic and epigenetic analyses, models of the epigenetic clock, and main results of the studies. Study characteristics are summarized in Table S1.

#### Assessment of study quality and risk of *bias*

With the help of the Critical Appraisal Checklist for Analytical Cross Sectional Studies of the Joanna Briggs Institute [32], the methodological quality of the studies was assessed. Because some of the studies also examined other variables than brain epigenetic age and included additional samples, the estimation of the risk of bias refers only to the outcome criteria, and not to the entire study.

### Evaluation of the quality of evidence

Evaluation of the quality of evidence was accomplished using the GRADE (Grading of Recommendations, Assessment, Development and Evaluation) manual. Study design, risk of bias, inconsistency, indirectness, imprecision of results, and possible publication bias were all considered [33]. Following the GRADE approach firstly evaluates the study design. Thereafter, the above criteria are considered, leading either to improved or worsened quality of evidence. Concerning study designs, randomized controlled studies possess a high quality of evidence. In contrast, observational studies possess a low quality of evidence. In the next step, the risk of bias is considered, and then the studies are checked for inconsistency (i.e., conflicting results). In the case of unexplainable inconsistencies, the quality of evidence is downgraded. Indirectness of results may result from differences between populations or interventions compared in a study, measures of only a proxy of the outcome of interest, or indirect comparisons between experimental and control groups. Afterwards, imprecision of studies is assessed. Imprecision may be due to small sample sizes and large confidence intervals. A further factor that may lower quality of evidence is publication bias [33]. A publication bias occurs in cases where relevant study results are not published, for example, because of non-significant results. This can lead to a misinterpretation of the evidence [34]. For each outcome (i.e., brain areas for each disease), the quality of evidence is determined separately. Four levels of quality can be achieved (high, moderate, low, and very low). These indicate the degree of confidence that effect estimates approximate real effects [35].

## Results

### Psychopathological entities

All studies featured a controlled cross-sectional design. Two studies examined bipolar affective disorder (BAD) [36, 37], three studies examined depression [38–40], one study examined posttraumatic stress disorder (PTSD) [40], one study examined obsessive compulsive disorder [41], five studies examined schizophrenia or schizoaffective disorder [42–45], and two studies examined alcohol addiction or alcohol abuse [40, 46]. Only one study included patients with different psychological disorders (i.e., PTSD, depression, and alcohol abuse) [40].

### Biomaterials and aging markers

All studies included brain specimens for epigenetic age examinations, whereby seven studies used exclusively brain tissues [36, 39–42, 44, 46]. Five studies also analyzed epigenetic age in peripheral blood [37, 38, 43, 45, 47], and one study also included liver tissue [47]. In two studies additional molecular aging markers were assessed, such as telomere length and the copy number of mitochondrial DNA per cell [36, 37]. In one study, DNA cytosine methylation was not assessed using DNA methylation chip arrays but instead by methylated DNA-binding protein (MBP)-seq [38]. MBP-seq is a method that involves the pulldown of methylated DNA using MBP, followed by deep sequencing to map DNA methylation patterns across the genome. One study, moreover, considered the klotho (KL) longevity gene polymorphism rs9315202 and examined a possible association of this genetic variation with epigenetic aging [40].

Six studies used original data from their own brain studies [36, 40–42, 46], and one of them, respectively, also from blood [38]. Two studies exclusively used publicly available brain datasets [39, 44], while another two used brain and blood datasets [43, 45]. Two studies used own original blood data in combination with public brain datasets [37, 47] and liver datasets [47] (Table S1).

### DNAm analyses and epigenetic clock models

Eleven of the studies used Horvath’s model in combination with different versions of Illumina’s Human DNA Methylation BeadChip Arrays (27K, 450K, EPIC) to determine epigenetic ages [26, 27]. Consequently, different but largely overlapping numbers of CpG sites were interrogated. Wu and co-workers further applied Hannum’s and Levine’s epigenetic clock models [28, 29] for their analyses [45]. Due to the excessive discrepancies between the epigenetic and chronological ages determined by these methods, the main results of this study relied exclusively on Horvath’s epigenetic clock. Only one study did not use BeadChip arrays [38]. Instead, this study used methyl-CpG-binding domain sequencing (MBD-seq). Therefore, the authors developed a custom epigenetic clock model, which they adapted and recalibrated using Horvath’s method. MBD-seq interrogates DNAm in a genome-wide manner. It is a precipitation method that uses MBD proteins to selectively capture CpG-methylated genome fragments, which thereafter undergo deep-sequencing. An overview of these studies, methodological characteristics, and main results can be found in Table S1 (see above, Sect. 3.3).

### Outcomes

#### Bipolar affective disorder

BAD has been examined in two studies [36, 37]. In their first study, the examination of epigenetic age in brain tissue was used to validate blood-derived findings. [37]. This study used publicly available cerebellar data from BAD patients and control subjects. No differences were found in the entire group or in subgroups divided by age. In 2020, the same research group conducted a study on hippocampal tissue from BAD-I patients and non-psychiatric control subjects [36]. Again, no significant acceleration in aging was found between BAD and control groups in the total sample. After splitting the samples into young and older individuals, a significant difference was found among the older subjects regarding accelerated aging. Furthermore, the group showed that epigenetic accelerated aging correlated negatively with telomere length and not at all with the number of mitochondrial DNA per cell. In another study with subgroups, no difference was found considering accelerated aging in BAD patients with and without suicide as a cause of death (Table 1).

**Table 1:** Summary of epigenetic age studies in various brain regions and mental disorders

Brain area	Disorder	Outcomes measured	Epigenetic age	Subjects		Study	Effect size measure
Frontal lobes				Number	Age Md, SD		
BA10, BA 25	MD	DNAm age blood, brain	MD on average + 1.1 years acc. DNAm age (*b* = 1.11, *χ2* = 3.41, *p* = .03)	74 MD, m/f: 42/32	54,1 ± 13,53	[38]	Cohen´s d = 0.18
				67 Controls, m/f: 39/28	57.03 ± 11.26		
PFC (BA 9)	AD AB	DNAm age blood, brain, liver	Trend, but no significance: dec. DNAm age in AD and AB (− .577); acc. DNAm age in control group (.577), respectively	23 AD/AB, m/f: 16/7 AD: 9	57.01 ± 9.26	[47]	N/A
				23 Controls, m/f: 16/7	56.04 ± 9.40		
Left motor cortex (BA 4)	PTSD MD AD/AB	Associative effects DNAm age; *KL* mRNA expression; KL regulation	Interaction effect between PTSD and acc. DNAm age in motor cortex of older subjects (*p* = .002*, p*_*corrected*_ = .014); acc. DNAm age in males with AD/AB in the motor cortex (*p* = .021)	*N* = 114, m: 61,2% PTSD: 40,5% MD: 69,0% AD/AB: 31,0% Controls: 24,1%	44,79 ± 13.93	[40]	SNP × PTSD on DNAm age residuals in motorcortex: Std β = 0.820* Alkohol abusus on epigenetic age in motor cortex: Std β = 0.221* Cohen´s d = 0.56
Frontal cortex	SZ	DNAm age blood, brain	dec. DNAm age in SZ group (*p* = .02); subgroup examination (by age-group): 20–39 yrs: significantly dec. DNAm age in SZ (*p *< .01); 40–59 yrs: trend towards dec. DNAm age in SZ; > 60 yrs: no difference	GSE74193 [61]; *N* = 675, f: 244		[45]	AcceleratorDiff decrease: Cohen´s q = 0.576 AccelerationRe-sidual increase: Cohen´s q = 0.464
				225 SZ	49.9 (17–97)		
				450 Controls	29.3 (0–85)		
				GSE61107 [62]; *N* = 47, f: 22			
				23 SZ	51.6 (20–94)		
				24 Controls	71.3 (53–90)		
				GSE61380 [63]; *N* = 33, f: 5			
				18 SZ	45.5 (24–73)		
				15 Controls	42.2 (21–69)		
				GSE128601[64]; *N* = 125, f: 54			
				75 SZ	53.1 (23–87)		
				50 Controls	59.3 (31–90)		
Left dlPFC (BA 9/46); left vmPFC (BA 12/32)	PTSD MD AD/AB	Interaction effects DNAm age; KL rs9315202 GT; *KL* mRNA expression; *KL* regulation	**No difference**	*N* = 114, m: 61,2% PTSD: 40,5% MD: 69,0% AD/AB: 31,0% Controls: 24,1%	44,79 ± 13.93	[40]	
PFC	MD	DNAm age brain	**No difference**	GSE88890 [65]; *N* = 40, f: 31		[39]	
				20 MD, m/f: 5/15	48.6 ± 20.8		
				20 Controls, m/f: 4/16	39.4 ± 19.5		
				GSE41826 [66]; *N* = 58, f: 30			
				29 MD, m/f: 14/15	32.5 ± 15.9		
				29 Controls, m/f: 14/15	32.6 ± 16.1		
dlPFC	SZ or SZA	DNAm age blood, brain	**No difference**	195 SZ or SZA, m/f: 119/76	Range: 17–96	[43]	
				232 Controls, m/f: 160/72	Range: 17–85		
PFC	SZ	DNAm age; Deviations of the methylome in group SZ	**No difference**	LNDBB; *N* = 43		[46]	
				20 SZ, m/f: 11/9	62.05 ± 15.87		
				23 Controls, m/f: 17/6	62.04 ± 18.74		
				DBCBB; *N* = 33			
				18 SZ, m/f: 15/3	45.50 ± 16.61		
				15 Controls, m/f: 13/2	42.27 ± 14.80		
dlPFC (BA 46/9)	SZ	DNAm age brain	**No difference**	[62]		[44]	
				24 SZ, m/f: 16, 8	52 ± 4.5		
				24 Controls, m/f: 19, 5	71.3 ± 2.0		
				[61]			
				175 SZ	50.24 ± 1.0		
				217 Controls	43.81 ± 1.0		
**Temporal lobe**							
Hippocampus	BAD I	DNAm age brain; mtDNA-CN; Telomere length	No difference in total sample; subgroup examination (by age-group/split at Md 46.6 yrs): acc. DNAm age among older (*p* = .042); acc. DNAm age negatively correlated with telomere length (r = -.337. *p* = .006); acc. DNAm age not correlated with mtDNA-CN	32 BAD I, m/f: 15/17	44.8 ± 13.7	[36]	Cohen´d for AA Subgroup analyses by age (Md 46.6 yrs) Cohen´s d = 0.798
				32 Controls, m/f: 19/13	46.9 ± 11.1		
Hippocampus	SZ	DNAm age blood, brain	**No difference**	67 SZ	54.0 (24–87)	[45]	
				75 Controls	55.1 (21–96)		
STG	SZ and SZA	DNAm age brain	**No difference**	22 SZ, m/f: 17/5 SZ: 16; SZA: 6	47.14 ± 2.91	[42]	
				22 Controls, m/f: 17/5	45,14 ± 2.30		
Hippocampus	SZ	DNAm age; Deviations of the methylome in SZ group	**No difference**	LNDBB; *N* = 27		[46]	
				14 SZ, m/f: 10/4	60.71 ± 15.93		
				13 Controls, m/f: 11/ 2	61.92 ± 17.80		
**Subcortical nuclei**							
Cortico-striatal: OFC, NAc, NuCa, PT	OCD	DNAm age; Differences in DNA methylation and gene expression	Trend towards acc. DNAm age; not significant	8 OCD, m/f: 5/3	76.4 ± 12.3	[41]	N/A
				8 Controls; m/f: 5/3	74.1 ± 13.6		
Cortico-striatal: ACC	OCD	DNAm age; Differences in DNA methylation and gene expression	Trend towards dec. DNAm age; not significant			[41]	N/A
STR	SZ	DNAm age; Deviations of the methylome in group SZ	**No difference**	LNDBB; *N* = 49		[46]	
				21 SZ, m/f: 11/10	61.76 ± 16.61		
				28 Controls, m/f: 20/8	63.43 ± 18.16		
				DBCBB; *N* = 33			
				16 SZ, m/f: 13/3	46.25 ± 17.10		
				17 Controls, m/f: 14/ 3	45.65 ± 16.82		
**Cerebellum**							
CER	BAD I	DNAm age blood, brain; mtDNA-CN; Telomere length	**No difference**	22 BAD I, m/f: 7/15	33.95 ± 9.3	[37]	
				20 Controls, m/f: 8/12	34.75 ± 10.0		
				16 siblings, m/f: 6,10	39 ± 10.6		
CER	SZ	DNAm age blood, brain	**No difference**	GSE38873 [67]; *N* = 102, f: 0		[45]	
				51 SZ	42.0 (20–60)		
				51 Controls	45.1 (30–70)		
				GSE61431 [63]; *N* = 87, f: 31			
				41 SZ	61.9 (31–87)		
				46 Controls	61.7 (25–96)		
CER	SZ	DNAm age; Deviations of the methylome in group SZ	**No difference**	LNDBB; N = 44		[46]	
				21 SZ, m/f: 11/10	61.76 ± 16.61		
				23 Controls, m/f: 17/6	61.39 ± 19.25		
				DBCBB; *N* = 33			
				16 SZ, m/f: 14/2	44.56 ± 15.84		
				17 Controls, m/f: 14/3	45.65 ± 16.82		

This table provides a summary of studies investigating epigenetic age alterations in different brain regions associated with various mental disorders. The table includes information on the brain area examined, the specific disorder studied, outcomes measured, epigenetic age alterations, subject characteristics, study details, and effect size measures. Abbreviations used in the table: *acc* accelerated (aging), *AB* alcohol abuse, *ACC* ventromedial prefrontal cortex, *AD* alcohol dependance, *BA* Brodmann area, *BAD* bipolar affective disorder, *CER* cerebellum, *dec* decelerated (aging), *DBCBB* The Douglas-Bell Canada Brain Bank, *dlPFC* dorsolateral prefrontal cortex, DNAm age epigenetic age measured by DNA methylation, *GEO* Gene Expression Omnibus, *GT* genotyping, *KL* gene klotho, *LNDBB* London Neurodegenerative Diseases Brain Bank, *MD* major depression, *mtDNA-CN* number of mitochondrial DNA per cell, *NAc* nucleus accumbens, *NuCa* nucleus caudatus, *OCD* obsessive–compulsive disorder, *OFC* orbitofrontal cortex, *PFC* prefrontal cortex, *PT* putamen, *PTSD* post-traumatic stress disorder, *f* female, *STG* superior temporal gyrus, *SZ* Schizophrenia, *SZA* Schizoaffective disorder, *STR* striatum, *vmPFC* ventromedial prefrontal cortex cell. (*Note: The values presented are in standardized beta (std. Beta) due to the unavailability of necessary data.)

#### Major depression

Depression has been examined in three studies [38–40]. Tissue from Brodmann area 11 as well as neurons and glial cells of the prefrontal cortex [39], Brodmann area 25 [38, 39], Brodmann area 10 [38], and Brodmann areas 9, 46, 12, 32, and 4 were examined [40]. Whereas Li et al. found no difference in the acceleration of epigenetic aging [39], Han et al. describe that the epigenetic age of patients with depression was on average 1.11 years higher than that of healthy subjects [38]. Wolf et al., who examined PTSD and alcohol addiction in addition to depression did not show results for depression [40] (Table 1).

#### Post-traumatic stress disorder

In addition to their analyses of depression, Wolf et al. examined the association between PTSD and epigenetic age in Brodmann areas 9, 46, 12, 32, and 4 and possible correlation with a klotho (KL) gene variant [40]. Their observations suggest a correlation between a KL SNP (rs9315202), PTSD and accelerated aging in the motor cortex in the elderly: Here, carriers of the rarer allele but not of the major allele were conspicuously often diagnosed PTSD and concomitantly exhibited accelerated epigenetic aging [40] (Table 1).

#### Obsessive compulsive disorder

De Oliveira et al. examined the correlation between obsessive compulsive disorder and epigenetic age in the anterior cingulate cortex, orbitofrontal cortex, nuclei accumbens and caudatus, and putamen [41]. While a trend towards slowered epigenetic aging was found in the anterior cingulate cortex, the other areas showed trends for accelerated aging, but neither trend was significant (Table 1).

#### Schizophrenia and schizoaffective disorder

Five studies examined the correlation between accelerated epigenetic aging and schizophrenia, more specifically, schizoaffective disorder. Considering that these studies partly used Gene Expression Omnibus (GEO) datasets, specimens used in three studies show at least partial overlap [43–45]. The areas examined were prefrontal cortex [43–46], striatum [45, 46], superior temporal gyrus [42], hippocampus and cerebellum [45, 46]. In their study, solely Wu et al. reported significant differences between the subjects with schizophrenia and the control group regarding epigenetic aging [45]. This study included the largest number of samples, but also overlapped with the other studies. The authors examined the prefrontal cortex, cerebellum, striatum, and hippocampus, but only found a difference in the frontal cortex, in terms of slowed epigenetic age. After a subgroup analysis, this difference remained significant only in the youngest group (20–39 years). In the midrange group (40–59 years), there was a trend toward slower aging in people with schizophrenia. No difference was found in people over the age of 60. Different areas of the prefrontal cortex were not investigated separately in this study (Table 1).

#### Alcohol addiction and abuse

Two studies examined alcohol addiction and alcohol abuse [40, 47]. Wolf et al. showed a significant correlation between alcohol dependence and accelerated aging in men in the motor cortex [40], whereas Rosen et al. showed slower epigenetic aging in Brodmann area 9 in the alcohol addiction or abuse group compared to the control group [47]. Fourteen of the 23 subjects were diagnosed with alcohol abuse, but not with alcohol addiction (Table 1).

## Discussion

In this structured review, we systematically analyzed studies on the putative role of epigenomic aging processes in patients with mental disorders and calculated effect sizes for the results reported in each study. We found that most studies used Horvath’s model of the epigenetic clock and either brain tissue or a combination of brain tissue with peripheral blood to assess markers of epigenetic aging. Schizophrenia and schizoaffective disorder, depression and bipolar disorder are the mental disorders, which were most frequently addressed in the studies included in the structured review. Brain regions most focused on were in the frontal cortex. Significant effect sizes were calculated for five studies targeting mainly frontal brain regions but different mental disorders. In these five studies, Horvath’s, and in one case Hannum’s and Levine’s epigenetic clock models were applied, and the results are based on highly varying sample sizes. To our knowledge, we present here the first structured review on studies on the role of epigenetic aging in mental disorders. We propose that our results may help to guide successful future methodological approaches to key functions of the epigenetic clock in different mental disorders.

### Brain regions

It is well known that most mental disorders are related to neurobiological aberrations in the prefrontal cortex [48–51]. Although distinct prefrontal dysfunctions are associated with each mental disorder [52], it is reasonable to assume that they all may be represented by markers of the epigenetic clock in frontal brain tissue [38, 53]. It is thus not surprising that most of the studies included in our structured review mainly addressed the frontal cortex to detect changes in the epigenetic clock in patients with mental disorders. Significant results were reported from authors who used frontal brain tissue for epigenetic analyses. In striatal, medial temporal, and cerebellar tissue, no significant associations between the epigenetic clock and mental disorders were found. Our data therefore suggest that tissue from different regions of the frontal cortex should be assessed systematically with distinct epigenetic methods to gain more specific insights into changes of the epigenetic clock related to mental disorders.

### Mental disorders

Schizophrenia and depression were targeted by most studies on the epigenetic clock in mental disorders. Depression is known to depend to a considerable extent on serotonergic and dopaminergic changes in the brain [54–56], whereas brain pathology underlying schizophrenia is more complex and also includes morphological brain abnormalities [57]. Despite these considerable differences, brain pathology related to both mental disorders is mainly located in the frontal cortex. Only [45] reported a significant difference in the frontal cortex for epigenetic aging between patients with schizophrenia and a healthy control group. These differences strongly depended on the age of subjects. In a subgroup analysis, epigenetic slowing was significant only in the youngest group (20–39 years). In the midrange group (40–59 years), there was a trend towards slower aging in patients with schizophrenia. No difference was found in people over the age of 60. These age differences may be related to aging-related neurodevelopment, developmental aspects of schizophrenia, duration of the disease, duration of medication, or interactions of all these factors [58]. This issue needs to be addressed in future studies of aberrant epigenetic aging associated with mental disorders. Notably, that the study by Wu et al. [45] included the largest sample sizes among those reviewed in this structured review. Different areas of the prefrontal cortex were not investigated separately in this study.

### Choice of methods

In the context of increasing numbers of CpG sites covered by DNA methylation BeadChip arrays, the number of statistical tests included in an ANOVA or other common statistical operations, also increases due to the increased number of variables (CpG sites). This gives rise to the well-known problem of alpha error accumulation. As the number of tests increases, the alpha error (false positive) also increases, resulting in a higher probability of finding a difference that is solely due to random variation. Consequently, the likelihood of erroneously rejecting the null hypothesis increases. The commonly used approach to correct for multiple comparisons is the Bonferroni approach. Alternative methods such as the family-wise error rate and the false discovery rate can be also employed. However, compared with the Bonferroni approach, they provide less stringent corrections and have a rather moderate impact on the study's power. Some of these corrections adjust the significance level downward based on the number of tests conducted. Instead of testing at a level of *p* < 0.05, for instance, a more stringent level like *p* < 0.0000005 may be employed when conducting a larger number of tests. This sets a very stringent threshold for significance. However, the strictness of these corrections introduces another issue. This is the reduction of study power due to controlling the alpha error. While the likelihood alpha errors is decreased by this method, the probability of making a beta error (falsely classifying a true effect as non-significant) increases. To mitigate this problem, one can increase the sample size to reduce the beta error again. It is important to note that this approach also has its limitations. When designing a study, the acceptable probabilities of errors must be carefully considered. In genomic research, one needs to be skeptical with respect to studies that utilize a large number of CpG sites, have a small sample size, and do not employ correction methods. We are here confronted with an acknowledged issue known as "p-hacking", which statisticians are well-aware of. This problem arises when authors, either intentionally or unintentionally, exploit calculated effects and argument with remarkable findings, which actually lack existence since they are solely attributed to random variation. Consequently, “p-hacking” has resulted in replication crises, especially in genomic research that utilizes DNA microarrays.

### Risk of *bias*

The risk of bias varied considerably between the individual criteria and studies. Low risk criteria were determined to be the clear inclusion criteria, the reliability and validity of the epigenetic age measurement method, and the statistical analyses. 83.33% of the studies showed high risk of bias due to failure to identify potentially confounding factors and inadequate approach to them. Only one-third of the studies showed a low risk of bias on the criterion of standardized and objective disturbance diagnostics. Due to a lack of information, the remaining studies had an uncertain risk in this criterion. Seven of the 12 studies showed a lower risk of bias in more than 50% of the criteria, whereas only two studies showed a low risk of bias in more than 80% of the criteria.

### Data synthesis and assessment of evidence quality

For each disease, a significant effect was found in at most one study and that only in specific brain areas or subgroup studies. Replications are needed to confirm the results. Table 1 shows the evidence profile of the individual outcomes with the assessment of evidence quality. A maximum of one study each showed significant results of accelerated aging in older BAD patients in the hippocampus (1/1), depression in the prefrontal cortex and subcortical areas (1/3), PTSD in the motor cortex with an interaction effect with SNP (1/1), alcohol addiction in men in the motor cortex (1/1), and significantly slowed aging in the frontal cortex in people with schizophrenia (1/4) and alcohol addiction (1/2). It is important to note that the quality of evidence for all outcomes must be classified as very low, which is due to the cross-sectional study design, an increased risk of bias, inconsistent, partly indirect results, small sample sizes and possible publication bias.

## Conclusions

Our structured review provides the first systematic overview of published data on the role of epigenetic aging in mental disorders, including the calculation of effect sizes for significant results of the reviewed studies. Results of our analyses show only a few significant effect sizes of published data. Importantly, however, we detected significant effect sizes for data from studies, which included large sample sizes, used both brain tissue and peripheral blood samples for epigenetic analyses, targeted markers of epigenetic aging in the frontal cortex, and focused on schizophrenia and depressive disorders. Both Horvath’s and Hannum’s and Levine’s epigenetic clock models proved to be valid conceptual bases for research on epigenetic aging in patients with mental disorders. Since data on the relevance of the age of patients for epigenetic aging is sparse, this issue needs to be investigated in detail in future studies on aberrant functions of the epigenetic clock in mental disorders. Moreover, the issue of putative therapeutic consequence from knowledge of changes in epigenetic aging in patients with mental disorders needs to be addressed. Controlled longitudinal clinical trials are required to clarify whether neuropsychological interventions, psychotherapy, and pharmacological treatment may reverse abnormal epigenetic aging associated with mental disorders.

The key advantage of epigenetic clock models is that it is not necessary to obtain biopsy material from the brain to measure the valid biological age of a subject. At the same time, however, one should be very aware that the measurement of a few hundred selected DNA methylation markers provides only a tiny insight into the epigenomic changes that most likely take place in the context of complex psychiatric diseases and gene regulation changes in the central nervous system.

We propose that the results of our structured review may help to guide successful future methodological approaches to key functions of the epigenetic clock in different mental disorders.

## Supplementary information

Below is the link to the electronic supplementary material.

Supplementary file1 Table S1. File ‘Table_S1.xls’ contains a technical comparison between the analyzed studies. This table provides a technical comparison of the studies analyzed in the research, focusing on various aspects such as the disorder studied, data sources, study groups, genotyping methods, DNA methylation analysis, mitochondrial DNA copy number assessment, telomere length evaluation, added sample information, epigenetic clock models used, number of CpGs analyzed, GEO datasets used, and sources of the studies. (XLSX 14 KB)

## Data Availability

All data generated during this study are results of analyses of previously published data sources. All data generated are included in this published article [and its supplementary information files].

## Abbreviations

ANOVA: Analysis of Variance
BAD: Bipolar Affective Disorder
CpG: Cytosine-phosphate-guanine
DNAm: DNA methylation
DNMTs: DNA methyl transferases
GEO: Gene Expression Omnibus
GRADE: Grading of Recommendations, Assessment, Development and Evaluation
KL: Klotho
MD: Major depression
OCD: Obsessive-compulsive disorder
PICO: Population, Intervention/Exposure, Comparison, Outcome
PRISMA: Preferred Reporting Items for Systematic Reviews and Meta-Analyses
PTSD: Posttraumatic Stress Disorder
SNP: Single Nucleotide Polymorphism
SZA: Schizoaffective disorder
WHO: World Health Organization
